# A Shift Toward Supercritical Brain Dynamics Predicts Alzheimer’s Disease Progression

**DOI:** 10.1523/JNEUROSCI.0688-24.2024

**Published:** 2024-12-30

**Authors:** Ehtasham Javed, Isabel Suárez-Méndez, Gianluca Susi, Juan Verdejo Román, J Matias Palva, Fernando Maestú, Satu Palva

**Affiliations:** ^1^Neuroscience Center, HiLIFE-Helsinki Institute of Life Science, University of Helsinki, Helsinki FI-00014, Finland; ^2^Center for Cognitive and Computational Neuroscience, Complutense University of Madrid, Madrid 28015, Spain; ^3^Department of Structure of Matter, Thermal Physics and Electronics, Complutense University of Madrid 28040, Spain; ^4^Department of Personality, Evaluation and Psychological Treatment, University of Granada 18071, Spain; ^5^Department of Neuroscience and Biomedical Engineering, Aalto University, Espoo 02150, Finland; ^6^Department of Experimental Psychology, Cognitive Processes and Logopedy, Complutense University of Madrid, Pozuelo de Alarcón 28223, Spain; ^7^Centre for Cognitive Neuroimaging, School of Psychology and Neuroscience, University of Glasgow, Glasgow G12 8QB

**Keywords:** brain criticality, detrended fluctuation analysis (DFA), excitation–inhibition imbalance, long-range temporal correlation (LRTC), machine learning, neuronal oscillations

## Abstract

Alzheimer’s disease (AD) is the most common form of dementia with continuum of disease progression of increasing severity from subjective cognitive decline (SCD) to mild cognitive impairment (MCI) and lastly to AD. The transition from MCI to AD has been linked to brain hypersynchronization, but the underlying mechanisms leading to this are unknown. Here, we hypothesized that excessive excitation in AD disease progression would shift brain dynamics toward supercriticality across an extended regime of critical-like dynamics. In this framework, healthy brain activity during aging preserves operation at near the critical phase transition at balanced excitation–inhibition (E/I). To test this hypothesis, we used source-reconstructed resting-state MEG data from a cross-sectional cohort (*N* = 343) of individuals with SCD, MCI, and healthy controls (HC) as well as from a longitudinal cohort (*N* = 45) of MCI patients. We then assessed brain criticality by quantifying long-range temporal correlations (LRTCs) and functional EI (fE/I) of neuronal oscillations. LRTCs were attenuated in SCD in spectrally and anatomically constrained regions while this breakdown was progressively more widespread in MC. In parallel, fE/I was increased in the MCI but not in the SC cohort. Both observations also predicted the disease progression in the longitudinal cohort. Finally, using machine learning trained on functional (LRTCs, fE/I) and structural (MTL volumes) features, we show that LRTCs and f/EI are the most informative features for accurate classification of individuals with SCD while structural changes accurate classify the individuals with MCI. These findings establish that a shift toward supercritical brain dynamics reflects early AD disease progression.

## Significance Statement

The neuronal mechanisms underlying progression of Alzheimer’s disease (AD) are not well understood. One characteristic feature of AD is brain hypersynchronization, but the mechanisms leading to this hypersynchronization have remained unclear. We investigated whether AD dementia progression can be explained in the framework of brain criticality by the shift in individual brain states along an extended regime of critical-like dynamics. We analyzed brain criticality with measures of long-range temporal correlations and functional excitation–inhibition balance from source-reconstructed resting-state magnetoencephalography data. We show that AD dementia progression is associated with a gradual increase in excitability and a progressive shift toward supercritical brain dynamics.

## Introduction

Alzheimer’s disease (AD) is a prevalent form of dementia, characterized by early memory loss and various cognitive impairments. AD progression follows a continuum of increasing severity from normal cognition to subjective cognitive decline (SCD), mild cognitive impairment (MCI), and lastly to AD (Jessen et al., 2014). Clinical AD diagnosis relies on physiological indicators such as amyloid-β (Aβ) plaques, changes in cerebrospinal fluid (CSF), p-tau, and structural atrophy in the medial temporal lobe (MTL), constituting ATN (amyloid, tau, neurodegeneration) axis (Jack et al., 2018; Zhang et al., 2021). Recent studies, however, link AD-related cognitive deficits to alterations in brain network connectivity in experimental (López-Sanz et al., 2017; Babiloni et al., 2020; Schoonhoven et al., 2022; Lorenzini et al., 2023) and simulated data (Zimmermann et al., 2018; Arbabyazd et al., 2021). Particularly, hypersynchronization of neuronal oscillations has been found to predict the progression from MCI to AD (Pusil et al., 2019; Maestú et al., 2021). This network dysfunction across AD continuum has been proposed to reflect progressive shift in the neuronal excitation–inhibition (E/I) ratio toward excessive excitation (Maestú et al., 2021; Ranasinghe et al., 2022; Giorgio et al., 2023).

**Figure 1.**
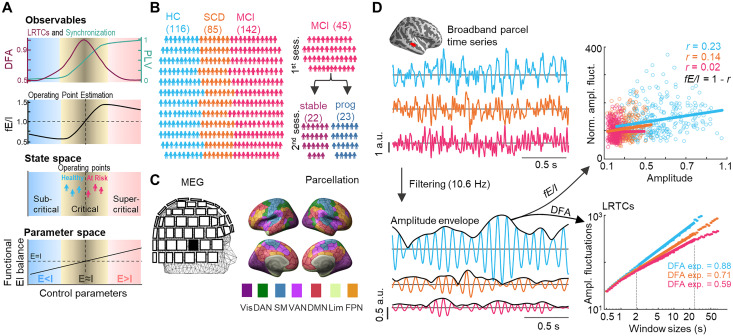
Hypothesis and approach. ***A***, Schematic of the brain criticality framework. The brain operates in an extended critical regime at the transition between sub- and supercritical phases. The critical transition point is characterized by a peak in the LRTCs and intermediate levels of synchronization and fE/I. Control parameters, such as E/I ratio, controls the operating point in the state phase. The critical regime is obtained with balanced E/I while excessive inhibition leads to operations in the subcritical and excessive excitation to supercritical regimes. ***B***, Left, Cross-sectional cohort size: 116 healthy controls (HC, blue), 85 participants with subjective cognitive decline (SCD, green), and 142 with mild cognitive impairment (MCI, orange). Right, Longitudinal cohort: 45 MCI, out of which for 22 patients’ symptoms remained stable and for 23 progressed to AD, at the follow-up. ***C***, Eyes-closed resting-state MEG data were recorded. MEG data was source-reconstructed and parcellated into Schaefer’s atlas. Colors reflect seven functional systems of the atlas. ***D***, Top left, MEG broadband time series from the superior temporal cortex for representative participants of each cohort (blue, HC; orange, SCD; and pink, MCI). Bottom left, Narrow alpha band (10.6 Hz) filtered parcel time series with their amplitude envelopes. Top right, Each dot in a scatter is a normalized amplitude fluctuation value corresponding to the mean amplitude. Values were calculated within time windows of which length equals to 40 cycles of filtering frequency. The fE/I is the linear relationship between these values. Bottom right, DFA exponents calculated from a slope of amplitude fluctuations as a function of window sizes in a log-log scale, for a representative parcel.

While it is impossible to directly assess the net E/I ratio in human brains by using noninvasive approaches, new promising approach enable inferring the E/I balance from “functional E/I” (fE/I) ratio and the slope of 1/f-like power spectra (Plenz and Thiagarajan, 2007; Shew et al., 2009; Gao et al., 2017; Bruining et al., 2020). These measures infer the E/I ratio in the brain criticality framework. Brain activity exhibits a 1/*f*-like power spectrum and spatiotemporally scale-free dynamics that are attributed to brain criticality (Plenz and Thiagarajan, 2007; He, 2014; S. Palva and Palva, 2018; O’Byrne and Jerbi, 2022). In this framework, healthy brains operate near a critical transition between subcritical and supercritical phases (states) in the system’s state space within an extended critical regime—the Griffiths phase (GP; Fig. 1*A*; Moretti and Muñoz, 2013; Villegas et al., 2014; Ódor and de Simoni, 2021; Fuscà et al., 2023). Operating within critical regime endows system with many functional benefits essential for healthy brain function as opposed to operating either in subcritical phase with attenuated activity propagation or in supercritical phase with amplifying activity, both functionally detrimental (Fig. 1*A*; O’Byrne and Jerbi, 2022; Fuscà et al., 2023). Importantly, the primary control parameter for tuning the operating point is E/I so that perfectly balanced E/I leads to operation at brain criticality (Villegas et al., 2014), whereas excitation-dominated E/I imbalance leads to supercriticality, as observed in AD patients (Montez et al., 2009; Martínez-Cañada et al., 2023; Van Nifterick et al., 2023). However, understanding the functional significance of excitation dominance in dementia progression has remained uninvestigated.

Here, we test the hypothesis that dementia progression would be associated with a gradual increase in excitability, shifting operating point away from balanced E/I regime toward supercritical-like dynamics. As a hallmark observables of brain criticality, local neuronal oscillation dynamics are characterized by neuronal avalanches (Beggs and Plenz, 2003; J. M. Palva et al., 2013; Zhigalov et al., 2015) and scale-free long-range temporal correlations (LRTCs), i.e., fluctuations over longer durations from seconds to minutes (Fig. 1*A*), with LRTCs being the strongest at the idealized critical point (Linkenkaer-Hansen et al., 2001; Kello et al., 2010; Poil et al., 2012). Thus, we hypothesized that shifting of the operating point within an extended critical regime (Fusca et al., 2023) from balanced E/I toward supercritical-like dynamics in dementia progression would be associated with progressive deterioration of LRTCs (Fig. 1*A*). Using large cross-sectional cohort (*N* = 343) of individuals with SCD, MCI, and healthy controls (HC) and a longitudinal MCI (*N* = 45) cohorts (Fig. 1*B*), we estimated fE/I (Bruining et al., 2020) and LRTCs (Linkenkaer-Hansen et al., 2001; Poil et al., 2012; J. M. Palva et al., 2013; Zhigalov et al., 2015) to assess the operating point and quantify brain criticality, respectively, for eyes-closed resting-state magnetoencephalography (MEG) data (Fig. 1*C*,*D*). We then used machine learning (ML) trained on functional (LRTCs, fE/I) and structural (MTL volumes) to classify early AD stages (SCD, MCI) to establish that these measures contain information of disease progression.

## Materials and Methods

### Cross-sectional and longitudinal cohorts

The cross-sectional dataset included 116 healthy controls (HC), 85 participants with subjective cognitive decline (SCD), and 142 with mild cognitive impairment (MCI; Fig. 1*B*, partially published dataset; Vaghari et al., 2022). The MCI participants (73.45 ± 5.44 years) were slightly older than the SCD participants (72.16 ± 5.29 years), who in turn were older than their HC peers (70.21 ± 4.38 years). Age differences, albeit small, were significant between HC and SCD participants (*t*_(199)_ = −2.86, *p* = 0.013, CI = [−3.31, −0.61], independent samples *t* test) and between HC and MCI participants (*t*_(256)_ = −5.21, *p* = 0.003 × 10–4, CI = [−4.48, −2.02], independent samples *t* test). There were no differences between groups in sex/gender (Table 1). Participants carried out a neuropsychological test battery including Boston Naming Test (BNT), Phonemic and Semantic Fluency Tests, Digit Span Test (forward and backward), and Logical Memory Test of the Wechsler Memory Scale-III (Spanish version; López-Sanz et al., 2017). SCD and MCI participants performed significantly (*p* < 0.05, uncorrected) worse than HC in most neuropsychological tests (Table 1). In each test, the performance of SCD participants lay within one standard deviation (SD) of the HC cohort and still within the range of performance considered “normal.” The MCI participants had much lower scores relative to the SCD and HC cohorts in all tests, objectively demonstrating impaired performance.

**Table 1.** Demographic and neuropsychological measurements

	HC	SCD	MCI
N	116	85	142
Age	70.21 ± 4.38*¶	72.16 ± 5.29*	73.45 ± 5.44¶
Gender (M/F)	39/77	18/67	50/92
BNT [0 60]	54.25 ± 6.56*¶	49.81 ± 7.62*^	44.22 ± 9.09¶^
Digit Span_F [0 16]	8.85 ± 2.56¶	8.43 ± 1.96^	6.97 ± 1.93¶^
Digit Span_B [0 14]	6.04 ± 2.01*¶	5.29 ± 1.78*^	4.27 ± 1.42¶^
LMIU [0 75]	40.90 ± 9.62*¶	33.54 ± 11.48*^	15.86 ± 8.84¶^
LMDU [0 75]	25.41 ± 7.49*¶	19.48 ± 8.72*^	5.78 ± 6.60¶^
LMIT [0 75]	16.98 ± 2.58*¶	15.21 ± 3.57*^	10 ± 4.14¶^
LMDT [0 75]	11.20 ± 1.99*¶	9.76 ± 2.96*^	4.08 ± 3.56¶^
Fluency Phonologic	14.93 ± 4.56*¶	11.49 ± 3.74*^	8.34 ± 4.06¶^
Fluency Semantic	17.31 ± 3.64*¶	15.39 ± 2.84*^	12.01 ± 3.37¶^
L hippocampal volume	0.0026 ± 0.0003*¶	0.0024 ± 0.0004*^	0.0022 ± 0.0004¶^
L hippocampal volume (reg)	0.0026 ± 0.0003*	0.0024 ± 0.0004^	0.0022 ± 0.0004¶^
R hippocampal volume	0.0025 ± 0.0003*	0.0025 ± 0.0004^	0.0022 ± 0.0004¶^
L parahippocampal volume	0.0014 ± 0.0003*	0.0014 ± 0.0003^	0.0012 ± 0.0002¶^
R parahippocampal volume	0.0013 ± 0.0002*¶	0.0012 ± 0.0002*^	0.0011 ± 0.0002¶^
R parahippocampal volume (reg)	0.0013 ± 0.0002*¶	0.0012 ± 0.0002^	0.0011 ± 0.0002¶^
L entorhinal volume	0.0013 ± 0.0002*¶	0.0013 ± 0.0003^	0.0011 ± 0.0003¶^
R entorhinal volume	0.0013 ± 0.0002*¶	0.0012 ± 0.0003^	0.0010 ± 0.0003¶^

The statistically significant differences between cohorts are highlighted for each cohort in the cross-sectional dataset.

The table shows (unless noted) mean ± standard deviation values of the demographic and neuropsychological measurements. Symbols (*, ¶, ^) denote a statistically significant difference (independent samples *t* test; *p* < 0.05; uncorrected) to another cohort: asterisk for HC–SCD, ¶ for HC–MCI, and ^ for SCD–MCI significant differences). Due to significant differences in age, we also looked at differences in cohorts in all variables after regressing out age confounds, which resulted in same statistics except for few mentioned explicitly with keyword “(reg)”. HC, neurotypical control; SCD, subjective cognitive decline; MCI, mild cognitive impairment; M, male; F, female; BNT, Boston Naming Test; F, forward; B, backward; LMIU, Logical Memory Immediate Units; LMDU, Logical Memory Delayed Units; LMIT, Logical Memory Immediate Thematic; LMDL, Logical Memory Delayed Thematic; L, left; R, right.

A subgroup of the subjects participated into a longitudinal study. At baseline, 142 participants diagnosed with MCI were engaged in the study and appointed for a longitudinal follow-up with cognitive and neuropsychological testing every 6 months (see below, Diagnostic criteria) with neuroimaging separated by ∼2 years. During this period, some participants were lost to follow-up. The longitudinal dataset here comprises 45 participants: 22 whose disease had not progressed to dementia and that were diagnosed with stable MCI (stable) and 23 whose disease progressed toward dementia with progressive MCI (prog) based on clinical assessments as described below. There were no significant differences in sex/gender. At baseline, there were no significant differences in age (stable: 71.73 ± 5.20; prog: 73.70 ± 3.40; *t*_(43)_ = −1.51, *p* = 0.138, *CI* = [−4.60, 0.66], independent samples *t* test) or MMSE score (stable: 27.45 ± 2.28; prog: 25.79 ± 3.34; *t* (43) = 1.88, *p* = 0.067, *CI* = [−0.12, 3.45], independent samples *t* test) between groups. In the post-session (time between recordings: stable: 2.33 ± 0.57 years; prog: 1.98 ± 0.85 years), there was a small but significant difference between groups in age (stable: 73.05 ± 4.85; prog: 75.87 ± 3.55; *t*_(43)_ = −2.22, *p* = 0.032, *CI* = [−5.39, −0.25], independent samples *t* test) and in MMSE (stable: 26.73 ± 3.06; prog: 23.41 ± 4.24; *t*_(43)_ = 1.88, *p* = 0.067, *CI* = [−0.12, 3.45], independent samples *t* test), which was expected since the duration of the follow-up depended on individual progression.

All the participants were right-handed, of Caucasian ethnicity, and native Spanish speakers. All participants signed informed consent before enrolling. The study was approved by the Ethics Committee of the Hospital Clínico Universitario San Carlos and conducted according to current guidelines and regulations.

### Diagnostic criteria

SCD diagnosis was determined using the SCD-I criteria: (1) self-reported cognitive concerns (mainly associated with memory); (2) normal performance on standardized cognitive testing; (3) feeling that the cognitive decline impairs everyday function; (4) seeking of medical consultation; and (5) age of 60 years or more at the onset of SCD (occurring within the last 5 years). The cognitive decline was also confirmed by a reliable informant. Definitive inclusion in this cohort was decided by multidisciplinary experts after discarding potential confounders of SCD (e.g., psychiatric/neurological disorders, medical conditions, prescribed drugs, or substance use). MCI diagnosis was determined using the NIA-AA guidelines for MCI due to AD (with intermediate likelihood; Albert et al., 2011): (1) self- or informant-reported cognitive concerns; (2) objective evidence of impairment in one or more cognitive domains; (3) preserved independence in everyday function; (4) absence of dementia, and (5) evidence of neuronal injury (hippocampal volume measured by MRI). Additional clinical tests were conducted to discard other potential causes of cognitive decline (e.g., B12 vitamin deficiency, diabetes mellitus, syphilis, and human immunodeficiency virus). Participants in the HC cohort were cognitive healthy and did not report cognitive complains. General exclusion criteria also involved (1) history of psychiatric/neurological disorders or drug usage that could influence MEG activity (e.g.,. choline esterase inhibitors); (2) evidence of infection, infarction, or focal lesions in a T2-weighted MRI scan within 2 months to the MEG recording (rated by two independent radiologist); (3) modified Hachinski score ≥5; (4) Geriatric Depression Scale–Short form score ≥5; and (5) history of alcoholism/chronic use of anxiolytics, neuroleptics, narcotics, anticonvulsants, or sedative hypnotics. For the diagnosis of longitudinal cohort, all MCI patients were followed up every 6 months for symptomatic and cognitive assessment. After a variable time period, some patients were classified as having developed dementia (here, referred to as progressive subcohort), according to the DSM-IV criteria or the 4–7 stages of the Reisberg Global Deterioration Scale (GDS) while others retained the diagnosis of MCI (stable subcohort). The appropriate classification of dementia as AD type was based on the National Institute of Neurological and Communicative Disorders and Stroke and the Alzheimer’s Disease and Related Disorders Association criteria (McKhann et al., 2011).

### Data acquisition

MEG data were recorded using a 306-channel (102 magnetometers, 204 planar gradiometers) Vectorview MEG system (Elekta Neuromag Oy) installed at the Center for Biomedical Technology. Before each recording, the participants were informed of the MEG routine, after which they voluntarily agreed to participate by signing an informed consent. The shape of their head was then digitally reproduced using a FASTRAK 3D digitizer (Polhemus). Specifically, ∼400 scalp points and three fiducial points (the nasion and the bilateral pre-auricular points) were digitized. In addition, four head position indicator (HPI) coils were applied to the scalp of the participants (two on the mastoids and two on the forehead) to monitor the position and movement of the head during the recording. Eyeblinks/movements and cardiac activity were also recorded using two pairs of electrodes arranged in bipolar montages. Another electrode, serving as a ground, was placed on the left earlobe. After the initial setup, the participants were instructed to sit quietly in the MEG system. Spontaneous electrophysiological activity was recorded for 3–5 min while the participants rested awake with their eyes closed. To prevent drowsiness during the recording, the participants received clear instructions on the importance of remaining alert throughout the experiment. A communication system was established to enable participants to promptly notify the experimenter of any feelings of drowsiness or fatigue, and additional queries were made at the end of the recording session. MEG data were sampled at Fs = 1,000 Hz with an online antialiasing bandpass filter between 0.1 and 330 Hz. Additionally, three-dimensional T1-weighted anatomical MRI scans were acquired in a 1.5 T MRI system (GE Healthcare) using a high-resolution antenna and a homogenization PURE filter (fast spoiled gradient echo sequence, with the following parameters: repetition time/echo time/inversion time, 11.2/4.2/450 ms; flip angle, 12°; slice thickness, 1 mm; 256 × 256 matrix; and field of view, 256 mm). MRI data were collected at the Hospital Clínico Universitario San Carlos (Madrid, Spain).

### MEG data preprocessing

The MEG data underwent a two-step preprocessing procedure using MATLAB and the FieldTrip toolbox (https://www.fieldtriptoolbox.org/; Oostenveld et al., 2011). Data preprocessing and analysis were conducted at two different sites, the Complutense University of Madrid (UCM; data preprocessing) and the University of Helsinki (UH; source reconstruction, parcellation, and all further data analysis steps). First, the temporally extended signal space separation method (tSSS) was applied using MAXFILTER (Elekta Neuromag Oy; correlation limit of 0.9, window length of 10 s) to remove external magnetic noises, interpolate bad channels, and compensate for head motion during the recording. Artifact detection was performed using the automatic artifact detection algorithm of the FieldTrip toolbox (UCM). Then, its output was visually inspected to discard false positives and to label additional undetected artifacts. The resulting muscular, ocular, and SQUID-jump artifacts were annotated, along with additional “high-amplitude” artifacts. For detected artifacts, the segments of artifacts were replaced with interpolated data accounted for only 2.24% of the total duration of the data. Of all segments of artifacts, 70.64% had a duration of <0.5 s, and 84.31% had a duration of <1 s. This was to preserve the original length of the recordings. Finally, an independent component analysis (ICA) procedure was applied to identify and clean the data from ocular and cardiac signals

### Source reconstruction and collapsed parcel time series

Source reconstruction was performed with minimum norm estimation (MNE) using dynamic statistical parametric maps (dSPM). The estimated dipole sources were then collapsed into Schaefer’s atlas of 400 parcels using a subject-specific fidelity weighting operator that weights sources by source reconstruction accuracy (Siebenhühner et al., 2020). Data were then filtered with 32 Morlet wavelets (width parameter *m* = 5) at log-equidistant spacing within range of 2–90 Hz.

### Analysis of functional excitation–inhibition and LRTCs

The functional excitation–inhibition ratio was estimated by using functional E/I (fE/I) as implemented in Bruining et al. (2020). Here, fE/I is defined as 1-*rW*_amp_, *W_n_*_(*F*(*t*)_ where *W* is Pearson’s correlation between the set of windowed detrended amplitude-normalized signal profiles, *w_nF(t)_*, and the set of windowed amplitude values, *w_amp_*. Inhibition-dominated networks will hence have *fE*/*I* < 1, excitation-dominated networks *fE/I* > 1, and at criticality, *fE*/*I* = ∼1. As recommended by Bruining et al. (2020), we included only signals with detrended fluctuation analysis (DFA) scaling exponents exceeding >0.6 into the fE/I analysis because the fE/I values are ambiguous further away from the critical region. The fE/I values were estimated for all cortical parcels from parcel time series for wavelet frequencies between 2 and 90 Hz.

The DFA exponents (Hurst exponents) were calculated with DFA (Linkenkaer-Hansen et al., 2001) as described previously (J. M. Palva et al., 2013; Zhigalov et al., 2015) which quantifies self-similarity, i.e., the strength of scale-free autocorrelations in the signal. The DFA scaling exponents >0.5 indicate the presence of significant power-law LRTCs.

DFA was computed for all cortical parcels from parcel time series for wavelet frequencies between 2 and 90 Hz. The fitting interval included window sizes from 2 to 25 s (with 50% overlap) and robust bisquare fitting yielded the estimated scaling exponent. The DFA exponents were then averaged across parcels to achieve mean DFA.

The mean fE/I and DFA exponents were obtained by averaging all parcel-level values across the whole brain for each wavelet frequency (Figs. 3*A*, 4*A*) or across parcels and frequencies within the alpha (7–12 Hz) and beta (12–30 Hz) bands (Fig. 5).

### Statistical analysis

The statistical significances of between-cohorts differences were examined at the whole-brain level for mean fE/I and DFA exponents using the nonparametric ANOVA (Kruskal–Wallis) between all three cohorts (HC, SCD, and MCI). Pairwise group differences between cohorts (HC–SCD, HC–MCI, SCD–MCI) were estimated at the whole-brain level for mean fE/I and DFA values and separately for each cortical parcel (parcel level). Statistical significances were assessed using the Wilcoxon test at *p* < 0.05 as well as *p* < 0.01. We used false discovery rate (FDR) correction with the Benjamini–Hochberg method to correct for multiple statistical comparisons, where the *p* values were adjusted by ranking them in ascending order, dividing by total number of statistical tests, and then multiplying by false positive rate (*q*) of 0.1 at whole brain level and 0.2 at parcel level. True positive significant findings are subsequently identified from adjusted *p* values using the alpha level of 0.05 or 0.01. The correlations between mean DFA and mean fE/I values with structural atrophy and neuropsychological assessment scores were estimated across subjects using Spearman’s correlation test.

### Machine learning analysis

We used ML analysis to test whether disease stage (HC, SCD, MCI) could be classified using the MEG-based (DFA, fE/I) structural (MTL volumes) features. Independent classifiers were implemented to dissociate the diagnostic cohorts in this study (HC vs MCI, HC vs SCD, and SCD vs MCI). The *k*-nearest neighbors (KNN) classification algorithm was used to classify individuals based on a set of MEG (LRTCs and *f*E/I) and structural (bilateral MTL volumes) features. The initial set of features included 42 functional MEG features, which were the DFA exponents and fE/I values averaged over those parcels with statistically significant effects and over wavelet frequencies in canonical frequency bands (theta, 4–7 Hz; alpha, 7–12 Hz; and beta, 12–30 Hz) within functional systems of the Schaefer atlas [default-mode network (DMN), dorsal attention network (DAN), frontoparietal network (FPN), limbic network (Lim), sensorimotor network (SM), ventral attention network (VAN), visual system (Vis)] which showed significant differences in pairwise comparisons in the parcel-level analysis. The six structural features included the bilateral MTL volumes (left and right hippocampi, parahippocampi, and entorhinal cortices). These features were extracted for each participant (Table 2). Data dimensionality was reduced by applying the feature selection algorithm *minimum-Redundancy-Maximum-Relevance* (mRMR; Radovic et al., 2017) to select a minimal-optimal subset of features. Classification models were trained on training sets using 75% of the data (seen) and the remaining 25% for testing (unseen). On the training data, we used the leave-one-out method, i.e., we repeated the training *n* times (where *n* = number of subjects in training data) with one subject left out of the training data. The best model obtained by this procedure was then evaluated on the unseen test data. Next, grid search was used for the selection of the three best models using the training data (seen) by varying the hyperparameter *k* of the kNN (10 < *k* < 20) and the number of features used (5 < *Nf* < 35). The model performance was estimated with using the receiver operating characteristic (ROC) curve. The three best models, one for each cohort-pair condition, as quantified having highest area under the curve (AUC) were used for testing classification performance (accuracy, precision, recall) on the test data (unseen). This use of separate test data not only demonstrates generalizability but also avoids any potential bias in classification accuracy due to feature selection (Shim et al., 2021).

**Table 2.** Consistency in classifier results using different set of features and classifiers

Metrics	Networks	Bands		Volumes	# features	
DFA, fE/I	FPN, DMN, DAN, Lim, VAN, SM VIs	Theta, alpha, beta		Left/right, Hipp. parahipp, entorhinal	48	
**Features importance for mRMR: HC-MCI**		**Features importance for mRMR: HC-SCD**			**Features importance for mRMR: SCD-MCI**	
R-Hipp, L-Hipp, R-ParaHipp, R-Ent, fE/I_θ_-Vis, fE/I_θ_-DAN, L-ParaHipp, DFA_α_-Vis, fE/I_θ_-FPN, fE/I_β_-SM, fE/I_β_-Lim, fE/I_α_-DAN, fE/I_θ_-DMN, DFA_θ_-Lim, DFA_β_-FPN, DFA_θ_-SM, DFA_θ_-SM		fE/I_β_-SM, fE/I_θ_-VAN, fE/I_β_-FPN, fE/I_β_-Vis, fE/I_θ_-SM, fE/I_α_-SM, DFA_α_-VAN, fE/I_β_-VAN, DFA_α_-SM, DFA_α_-Lim, DFA_α_-FPN, fE/I_α_-DAN, DFA_α_-DMN, DFA_α_-DAN, fE/I_θ_-DMN, fE/I_α_-Vis, DFA_α_-SM, fE/I_θ_-Vis, DFA_β_-Lim, DFA_β_-DMN, DFA_β_-SM, fE/I_θ_-Lim, DFA_β_-Vis, DFA_θ_-SM, DFA_β_-VAN, DFA_β_-DAN, DFA_θ_-Vis, fE/I_θ_-DAN, DFA_θ_-DMN, DFA_β_-FPN, DFA_θ_-Lim, DFA_θ_-FPN, DFA_θ_-VAN			L-Hipp, fE/I_α_-Lim, fE/I_θ_-DMN, R-Ent, fE/I_θ_-Lim, fE/I_β_-Vis, DFA_θ_-SM	
**Performance for fixed 10 features for all kNN classifiers**						
**Classes (*n* trials)**	**Hyperparameter fixed features**	**Acc.**	**Bal. Acc.**	**Precision**	**Rec.**	**AUC**
HC, MCI 87, 107 (29,35)	*K* = 13 M + 3S	0.82 (0.78)	0.82 (0.79)	0.83 (0.83)	0.85 (0.84)	0.86 (0.81)
HC, SCD 87, 64 (29,21)	*K* = 11 10 M	0.72 (0.70)	0.70 (0.68)	0.70 (0.69)	0.58 (0.52)	0.76 (0.70)
SCD, MCI 64, 107 (21,35)	*K* = 19 6M + 4S	0.76 (0.73)	0.71 (0.67)	0.76 (0.73)	0.91 (0.91)	0.83 (0.83)
**Performance for all SVM classifiers**						
**Classes (*n* trials)**	**Hyperparameter fixed features**	**Acc.**	**Bal. Acc**	**Precision**	**Rec.**	**AUC**
HC, MCI 87, 107 (29,35)	12M + 5S	0.86 (0.77)	0.86 (0.77)	0.93 (0.81)	0.79 (0.74)	0.91 (0.80)
HC, SCD 87, 64 (29,21)	33M	0.75 (0.74)	0.74 (0.72)	0.72 (0.72)	0.66 (0.62)	0.78 (0.75)
SCD, MCI 64, 107 (21,35)	7M + 4S	0.76 (0.73)	0.74 (0.68)	0.81 (0.74)	0.81 (0.89)	0.82 (0.69)
**Performance for fixed 10 features for SVM classifiers**						
HC, MCI 87, 107 (29,35)	7M + 3V	0.80 (0.75)	0.80 (0.76)	0.82 (0.85)	0.83 (0.66)	0.85 (0.78)
HC, SCD 87, 64 (29,21)	10M	0.68 (0.66)	0.67 (0.67)	0.61 (0.61)	0.66 (0.66)	0.73 (0.73)
SCD, MCI 64, 107 (21,35)	6M + 4V	0.78 (0.75)	0.76 (0.70)	0.82 (0.74)	0.82 (0.91)	0.83 (0.71)

Feature matrix and importance of different features are shown for mRMR, kNN, and SVM classifiers.

We used Shapley additive explanations (SHAP) plots to explore the contributions of the different features to the predictions (Aas et al., 2021). The classifications carried out with the kNN were repeated using the support vector machine (SVM) method (using a Gaussian kernel). The best classifiers for classifying HC versus MCI, HC versus SCD, and SCD versus MCI using 10 features were also selected using above mentioned grid search.

### Data availability

The raw data analyzed during this study are stored at a secure UCM server accessible at the URL https://vbc.ucm.es/login.php. Access can be granted by S.P. and F.M. upon written request along with the clarification as to what purpose the data will be used. The processed data will be made publicly available upon publication at https://datadryad.org/stash/dataset/doi:10.5061/dryad.wwpzgmspw. The custom code written with MATLAB (R2017b, MathWorks), which reproduces this manuscripts’ central results and conclusions is openly available at https://github.com/palvalab/EI-unbalance-and-DFA-exponents.

## Results

### Clinical demographics

**Figure 2.**
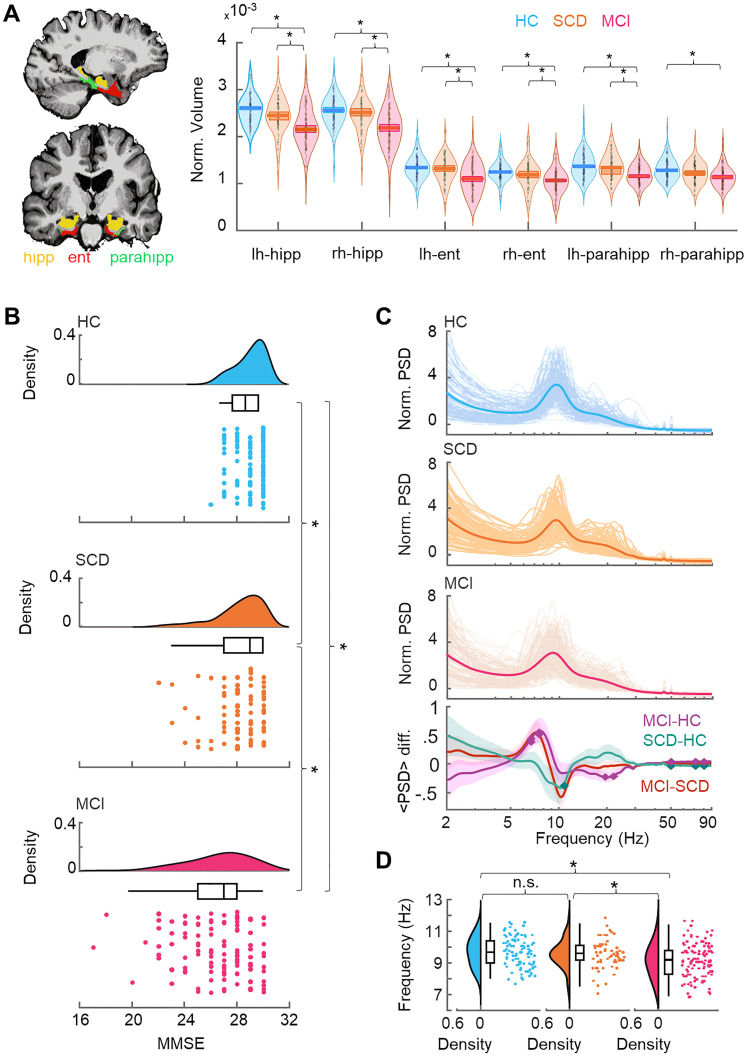
Structural and spectral differences between cohorts. ***A***, Pairwise comparison shows reduced volume (* denotes *p* < 0.05; Wilcoxon test; Bonferroni corrected; *N_test_* = 18) in MCI compared with HC and SCD cohorts in regions of the medial temporal lobe (MTL; hippocampal, entorhinal and parahippocampal). No significant differences were observed between HC and SCD. ***B***, MMSE scores for HC, SCD, and MCI cohorts. MMSE scores were different (* denotes *p* < 0.05; Wilcoxon test; Bonferroni corrected; *N_tests_* = 3) between cohorts. Individual participants’ MMSE score with a boxplot denoting the first, median, and third quartiles, and the whisker lengths representing 1.5 times the interquartile range. ***C***, Normalized power spectral density (PSD), averaged across parcels and within cohorts. Bottom, Pairwise between cohort differences [Wilcoxon test; *p* < 0.05; false discovery rate (FDR) corrected; *N_tests_* = 32]. ***D***, Peak alpha frequencies plotted as in ***B*** with significant (*p *<* 0.05; independent samples *t* test; Bonferroni corrected with *N_tests_* *=* 3) and nonsignificant (n.s.) between cohort differences.

The clinical diagnosis of AD dementia is generally obtained through validated neuropsychological tests and currently also using biomarker evidence such as MTL atrophy to obtain a reliable diagnosis (Hampel et al., 2022). Following this procedure for the participants of the present study, we first assessed the diagnostic criteria (see Materials and Methods, Diagnostic criteria) for mental decline using MMSE tests and for MTL volumes using anatomical MRIs. MCI participants showed smaller volumes in all bilateral MTL regions (hippocampus and entorhinal cortex) than SCD and HC participants (Fig. 2*A*; *p* < 0.05; Wilcoxon test; Bonferroni corrected; *N_tests_* = 18). MMSE scores were also significantly lower in SCD and MCI compared with those in HC and lower in MCI compared with those in SCD cohorts (Fig. 2*B*; *p* < 0.05; Wilcoxon test; Bonferroni corrected; *N_tests_* = 3).

At baseline, 142 participants diagnosed with MCI were further engaged in a longitudinal study where their diagnosis was assessed every 6 months for a maximum of 4 years and a second neuroimaging recording was performed after 2 years. After assessing their clinical status at this follow-up, these MCI patients were divided into stable and progressive subcohorts depending on whose disease had not (stable) or had (progressive) evolved toward more severe dementia based on neuropsychological assessments (see Materials and Methods). During longitudinal study, 29 participants with probable stable MCI and 21 participants with probable AD dropped out. We did not find significant differences in cognitive performance between participants who completed the study or dropped out, but the possibility of subtle biases related to dropout cannot be fully discarded.

Of the remaining participants with MEG data used in this study, 22 participants were diagnosed with stable MCI while 23 had converted from MCI to probable AD (progressive MCI). The separation of 1st and 2nd MEG measurements was 1.98 ± 0.85 years for progressive and 2.33 ± 0.57 years for stable cohort. The progressive subcohort had significantly worse MMSE scores at second measurement compared with the stable subcohort (stable: 26.73 ± 3.06; prog: 23.41 ± 4.24; *t*_(43)_ = 2.98, *p* = 0.005, *CI* = [1.07, 5.57], independent samples *t* test) and to their baseline performance wherein their diagnoses did not differ [prog (1st measurement): 25.79 ± 3.34; prog (2nd measurement): 23.41 ± 4.24; *t*_(22)_ = 4.08, *p* = 0.0008, *CI* = [0.75, 2.36], paired *t* test]. Conversely, there were no significant differences on stable participants’ MMSE scores across sessions [stable (1st measurement): 27.45 ± 2.28; stable (2nd measurement): 26.73 ± 3.06; *t*_(21)_ = 1.34, *p* = 0.195, *CI* = [−0.40, 1.86], paired *t* test].

### Oscillation power changes

We assessed the amplitudes of neuronal oscillations with the classical power spectral density (PSD) approach (Fig. 2*C*). PSD has earlier been found to differ between HC and MCI patients (Garcés et al., 2013; Jiao et al., 2023). Here, we found significant PSD differences in the alpha band (8–12 Hz) such that both in the HC–MCI and SCD–MCI comparisons, there was reduced PSD at ∼10 Hz, while in 7 − 8 Hz in MCI and SCD suggestive of MCI being characterized by a shift of the PSD alpha peak at toward lower frequencies (Fig. 2*C*, bottom panel) as has been found earlier (Jiao et al., 2023). We corroborated this finding with a peak-frequency analysis that also revealed a significant slowing of alpha-band oscillations (Fig. 2*D*; *p* *<* 0.05; independent samples *t* test; Bonferroni corrected with *N_tests_* *=* 3). However, in line with prior studies, we did not find HC–SCD power differences or a shift of the alpha PSD peak toward lower frequencies for the SCD cohort at the whole brain level for mean PSD (Fig. 2*C*,*D*).

### MCI is characterized by a shift toward greater excitability in fE/I

We then investigated whether the disease progression across the HC–SCD–MCI continuum would be associated with altered excitation–inhibition ratio (E/I) by using a new measure of functional E/I (fE/I; Bruining et al., 2020). This measure is an estimate of the operating point of the neuronal systems in the state space around a critical phase transition between subcritical and supercritical phases. As the primary control parameter determining this operating point is thought to the E/I ratio (Plenz and Thiagarajan, 2007; Shew et al., 2009), a shift in the fE/I metric is considered to reflect changes in E/I and the level of excitability.

**Figure 3.**
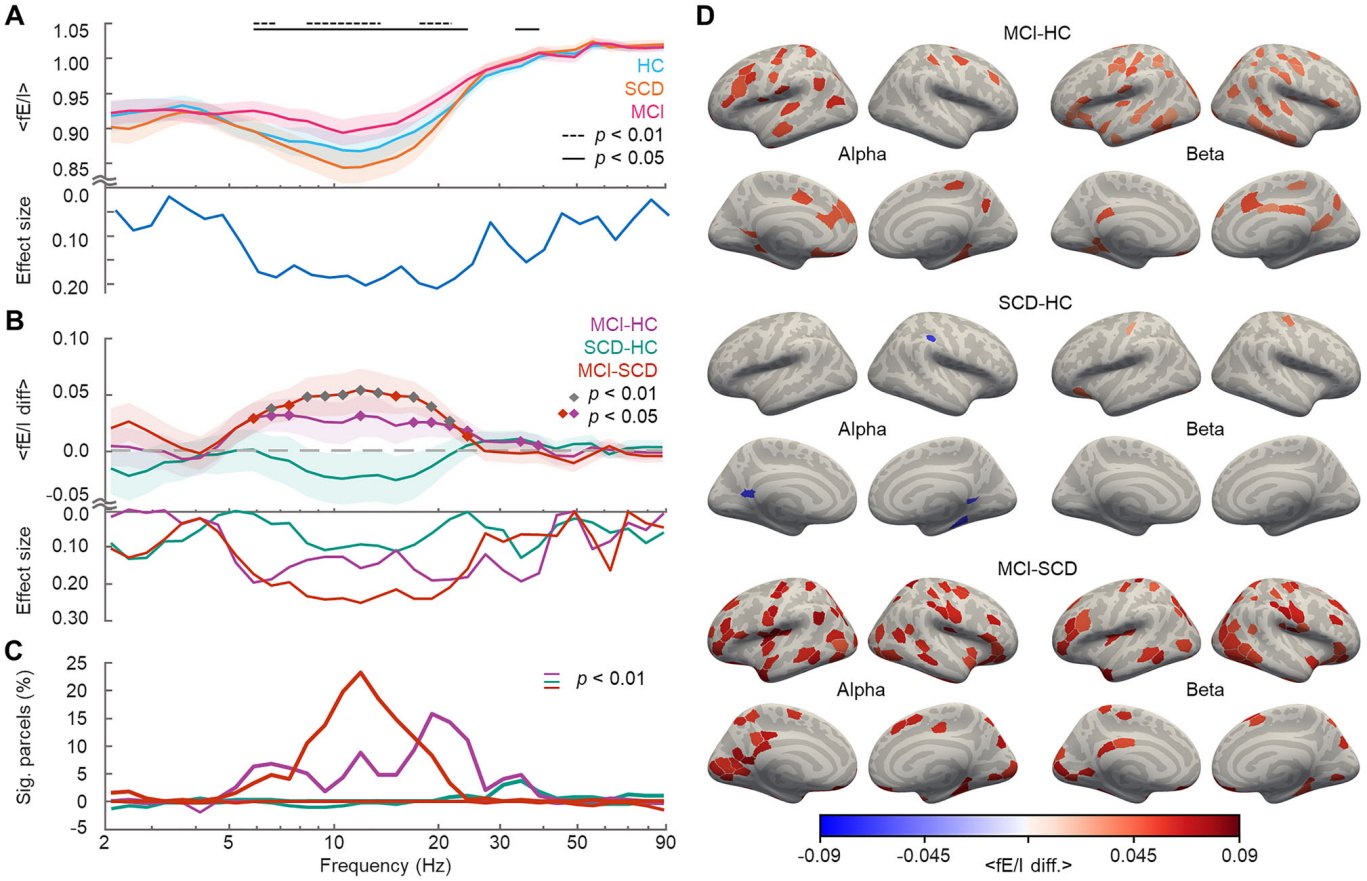
The E/I balance shifts toward greater excitability along the disease trajectory from HC to MCI. ***A***, Mean fE/I values for each cohort (cyan, HC; orange, SCD; and pink, MCI) averaged over all parcels. Shaded areas indicate 95% confidence intervals calculated using bootstrapping (*n* = 10,000) method. The line indicates the frequencies with significant *p* < 0.05 and dashed line *p* < 0.01 differences across cohorts (Kruskal–Wallis test; FDR corrected; *N_tests_* = 32). ***B***, Pairwise differences between the cohorts in the mean fE/I (purple, MCI–HC; green, SCD–HC; and brown, MCI–SCD). Shaded areas indicate 95% confidence intervals. The diamonds mark the frequencies with significant (*p* < 0.01 and 0.05; Wilcoxon test) differences. ***C***, The percentage (%) of parcels, which show significant positive or negative differences in fE/I between the cohorts as in ***B*** in the parcel-level analysis. ***D***, Between cohort differences for mean fE/I across alpha and beta band in cortical anatomy.

At the whole brain level, we found mean fE/I values ranging from ∼0.85 to 0.95, the individual mean fE/I values being significantly different across cohorts (Fig. 3*A*; Kruskal–Wallis test; *p* < 0.05 and *p* < 0.01; FDR corrected; *N_tests_* = 32; effect size, *η*^2^ > 0.02) throughout the alpha to gamma (7–40 Hz) frequency bands. Subsequent post hoc pairwise tests between the cohorts revealed that these differences were due to a significant increase in *f*E/I values in the MCI relative to the HC cohort between 5 and 25 Hz (Fig. 3*B*; Wilcoxon test; *p* < 0.05 and *p* < 0.01; *r* > 0.17) and thus a shift toward greater excitability in MCI. The mean fE/I values were intriguingly smaller in SCD than those in HC cohort, but this difference did not reach statistical significance implying that excitability shifts appear only at a later stages of the disease progression. Nonetheless, given that AD is associated with structural changes that initially only take place in a small subset of brain regions (Jack et al., 2018), we also assessed the fE/I differences (Wilcoxon test; *p* < 0.01; FDR corrected, *N_tests_* = 38,400) between the cohorts at the parcel level (Figs. 3*C*). Consolidating the whole brain results, both HC–MCI and SCD–MCI differences were significant also at the parcel level, but with different spectral profiles. MCI–HC difference was present from 5 to 35 Hz, being most robust in the beta band (17–25 Hz). In contrast, MCI–SCD difference was present and robust from 5 to 25 Hz, being most extensive at ∼10 Hz suggesting a frequency shift of increased excitation from alpha to beta frequency as a function of disease progression. These differences were found in widespread cortical structures (Fig. 3*D*).

### Attenuated LRTCs characterize SCD and MCI stages

**Figure 4.**
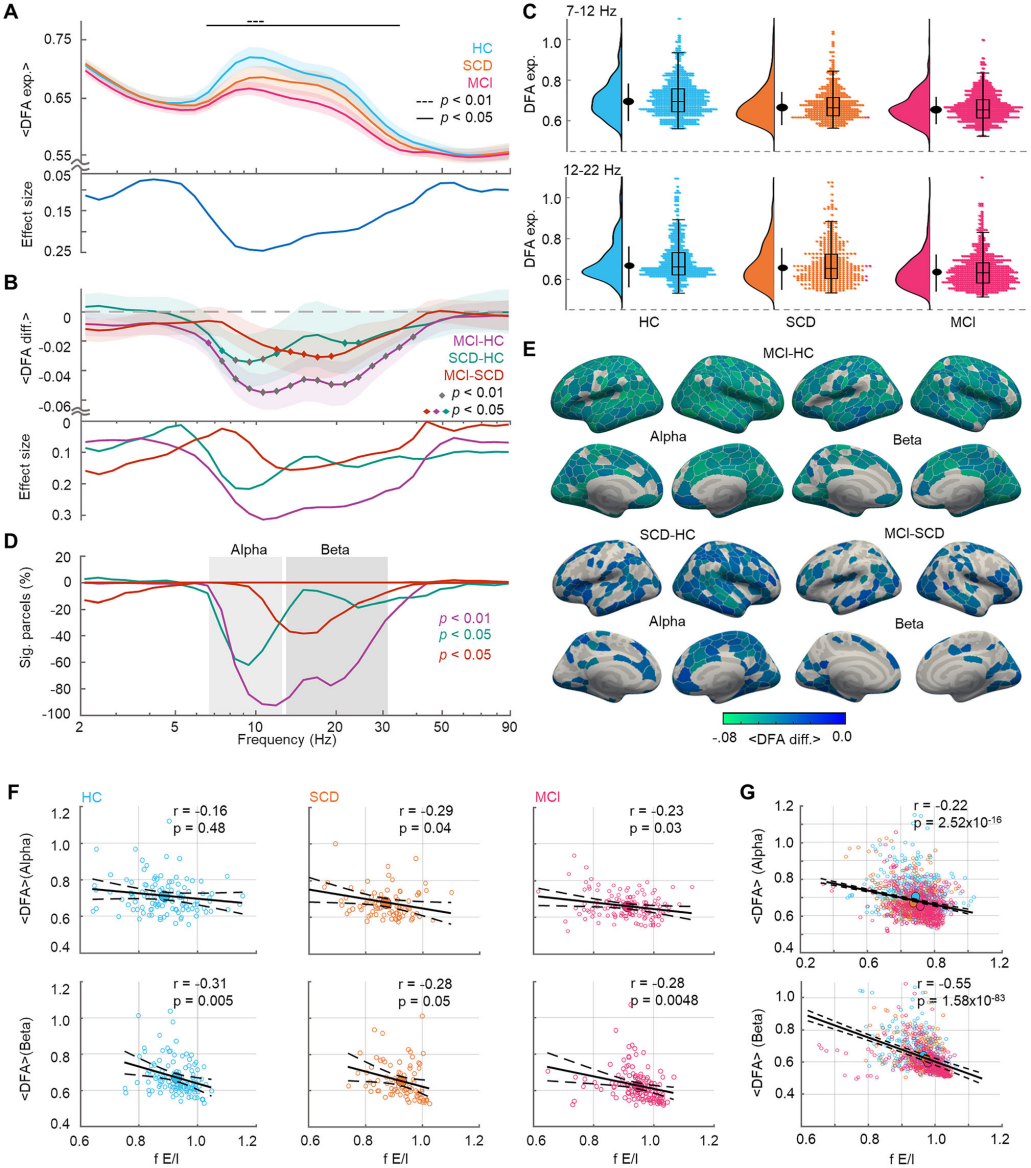
LRTCs dissociate HC, SCD, and MCI cohorts. ***A***, Mean DFA exponent, averaged across parcels and within cohorts. Shaded areas represent bootstrapped (*N* = 10,000) 95% confidence intervals. Significance as in Figure 3*A*. ***B***, Pairwise differences between cohorts in the mean DFA exponents. The diamonds highlight significant differences (*p* < 0.01 and *p* < 0.05; Wilcoxon test). ***C***, Density plots for mean DFA exponents averaged within alpha (7–12 Hz) and beta (12–22 Hz) bands. The black-filled dot denotes median, and the line length represents the standard deviation. Individual participants’ DFA exponents (right) with an overlaid boxplot denoting the first, median, and third quartiles, and the whisker lengths representing 1.5 times the interquartile range. ***D***, The percentage of parcels, which show significant positive or negative (Wilcoxon test; *p* < 0.01 or *p* < 0.05; FDR corrected; *N_tests_* = 38,400) differences in DFA exponents between cohorts. ***E***, Mean DFA differences between the cohorts averaged within alpha (7–12 Hz) and beta bands (12–30 Hz) bands. ***F***, Correlation of mean alpha and beta DFA exponents and fE/I values for HC, SCD and MCI cohorts. ***G***, Correlation of mean alpha and beta DFA exponents and fE/I values pooled across all subjects.

To supplement the operating point estimates with a more established measure of brain criticality, we quantified the strengths of emergent power-law LRTCs in oscillation amplitude fluctuations with DFA (Hardstone et al., 2012). DFA exponents describe signal’s self-similarity and quantify the strength of LRTCs so that large exponents indicate stronger dependencies over time. LRTCs peak at criticality and approach white-noise levels both toward inhibition-dominated subcritical and excitation-dominated supercritical states (Linkenkaer-Hansen et al., 2001; Poil et al., 2012; J. M. Palva et al., 2013; Fuscà et al., 2023). The HC cohort exhibited a broad peak in alpha to beta frequencies (7–30 Hz) in the mean DFA exponent at the whole-brain level (Fig. 4*A*) indicating a preservation of critical-like brain dynamics in the elderly cohort. LRTCs were, however, significantly different between HC, SCD, and MCI cohorts between 7 and 40 Hz (Fig. 4*A*; Kruskal–Wallis; *p* < 0.05 and *p* < 0.01; FDR corrected; *N_tests_* = 32; effect size: *η*^2^ > 0.02 for significant observations). Pairwise post hoc test between the cohorts revealed a salient progressive breakdown of LRTCs with dementia progression. Significant HC–SCD differences were found in the alpha band (7–12 Hz) while SCD–MCI differences expanded to beta band (12–22 Hz) both for each wavelet frequency (Figs. 4*B*) and when averaged over the frequency bands (Figs. 4*C*).

Also at the parcel level, DFA exponents between the cohorts differed in a large fraction of the parcels; HC–MCI cohorts in the alpha-beta bands, HC–SCD in the alpha band ,and SCD–MCI in the beta band [Fig. 4*D*; Wilcoxon test; *p* < 0.01 (HC–MCI); *p* < 0.05 (HC–SCD; SCD–MCI); FDR corrected; *N_tests_* = 38,400; effect size, *r* > 0.14]. Neuroanatomically, the HC–MCI differences were widespread and observed in all functional systems and parcels while the HC–SCD and SCD–MCI differences were more constrained (Fig. 4*E*) suggesting a spreading of neuronal dysfunction. To validate that fE/I and DFA exponents reflect the same underlying construct of the operating point in the critical dynamics state space, we tested for their correlation across individuals (Fuscà et al., 2023) and found negative linear correlations for all cohorts (Fig. 4*F*; Pearson’s correlation coefficient; *p* < 0.05; Bonferroni corrected). Correlation values for HC and MCI cohorts were also separable in state space (Fig. 4*G*), supporting the hypothesis that the operating points in the critical state space for these two cohorts are different.

### Brain criticality is correlated with MTL volumes and neuropsychological performance

**Figure 5.**
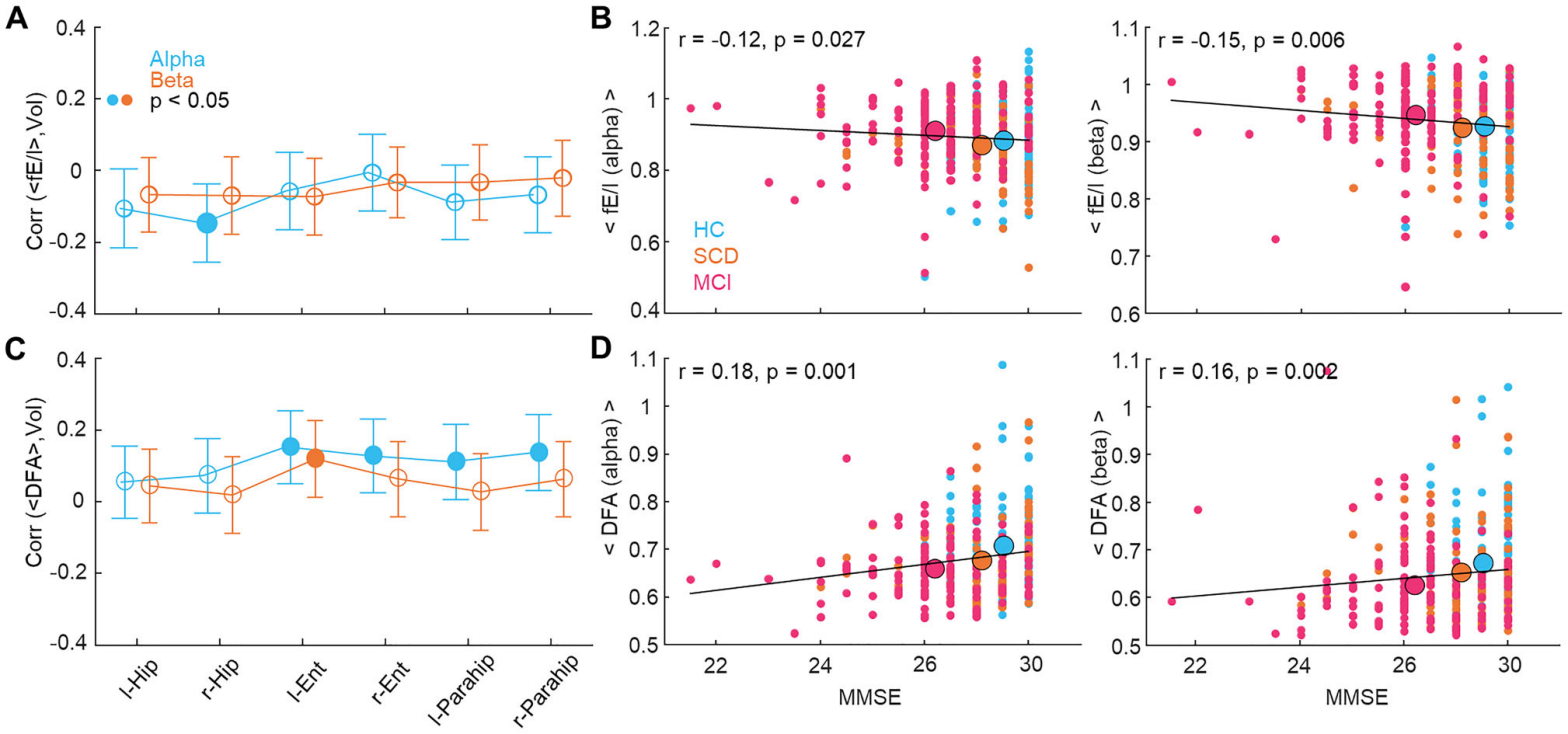
Aberrant LRTCs are correlated with structural and cognitive deficits. ***A***, Correlation across all participants (Spearman; solid dots, *p* < 0.05; shallow dots, not significant) of the mean fE/I values averaged across parcels for each participant with individual bilateral MTL structural volumes (l, left; r, right; Hip, hippocampus; Parahip, parahippocampus; Ent, entorhinal cortex) and (***B***) with MMSE. Bars represent bootstrapped (*N* = 10,000) 95% confidence intervals. ***C***, Correlation across all participants of mean DFA values with MTL volumes and (***D***) with MMSE as in ***A*** and ***B***.

The clinical diagnosis of probable AD dementia is generally achieved through validated neuropsychological assessments but increasingly emphasizes the use of biomarkers such as neuronal injury (MTL volumes measured by MRI) to enhance the diagnostic accuracy (Hampel et al., 2022). We investigated whether mean fE/I DFA exponents averaged across parcels would be correlated with individual MTL volumes and neuropsychological performance (MMSE) across subjects. There was a significant negative association (Spearman correlation; filled dots: *p* < 0.05) between alpha-band mean fE/I and right-hippocampal volume. Similarly, in both alpha- and beta-frequency bands, mean fE/I values were negatively correlated with cognitive performance, showing that elevated fE/I values are associated with worsening of cognitive performance (Fig. 5*B*). Conversely, DFA exponents averaged across parcels for alpha band were positively correlated with bilateral entorhinal (left: *r* = 0.15, *p* = 0.0045; right: *r* = 0.13, *p* = 0.0173), and parahippocampal volumes (left: *r* = 0.11, *p* = 0.0383; right: *r* = 0.14, *p* = 0.0110), and between beta-band mean DFA exponents and left entorhinal volume (*r* = 0.12, *p* = 0.0261), indicating that greater DFA exponents, which characterize healthy controls, were associated with greater MTL volumes (Fig. 5*C*).

Similarly, in both alpha- and beta-frequency bands, mean DFA exponents were positively correlated with MMSE when age was used as a covariate showing that stronger LRTCs are associated with better cognitive performance (Fig. 5*D*). Overall, these results confirmed that multiple measures of brain criticality were associated with MTL volumes or neuropsychological performance traditionally used in clinical AD diagnosis, albeit with correlation of limited magnitude.

### LRTCs and fE/I differ for stable and progressive MCI patients

**Figure 6.**
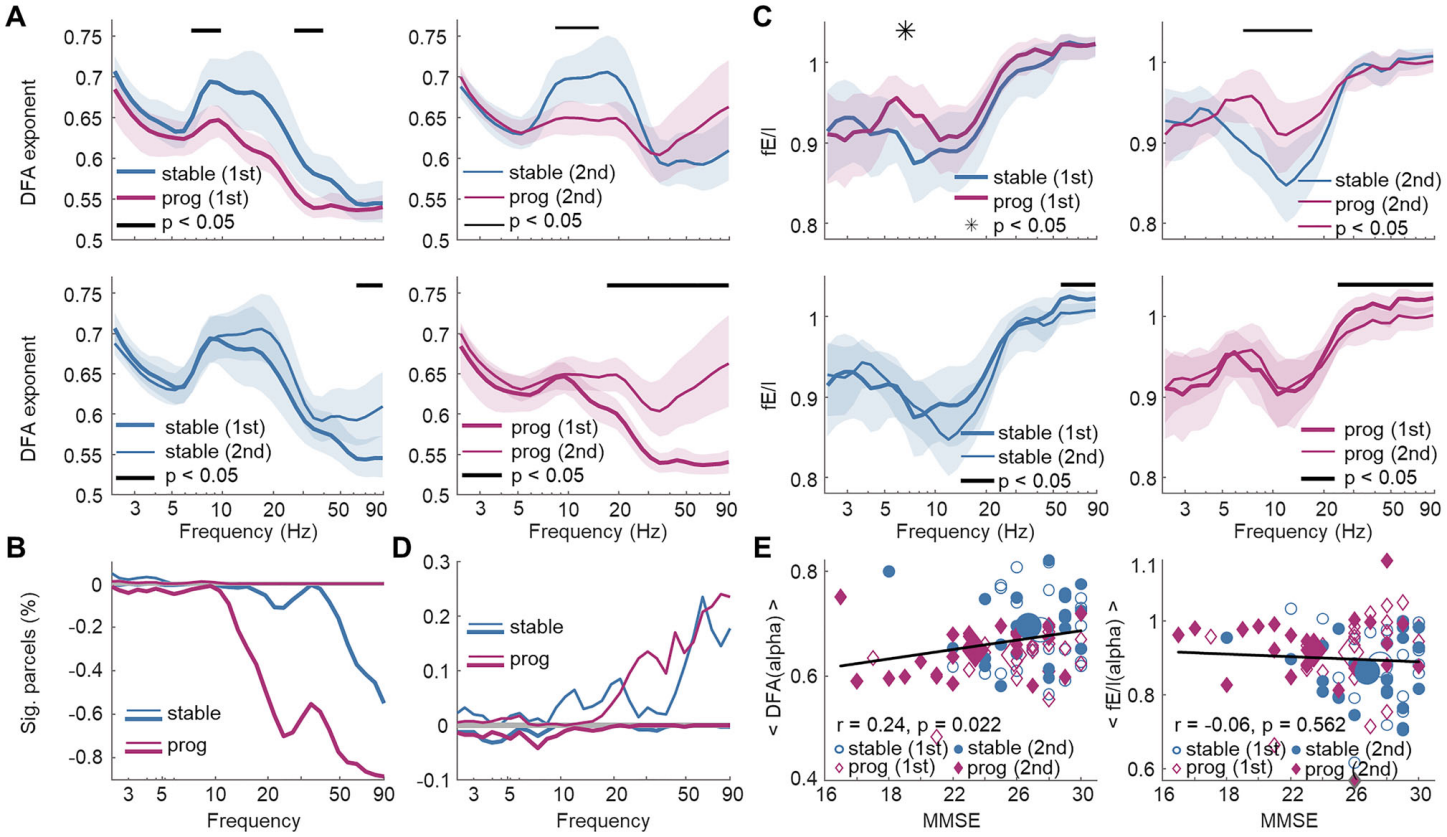
Longitudinal data: aberrant LRTCs and fE/I predict disease progression. ***A***, Mean DFA exponent differ between stable and progressive MCI cohorts (stable, *N* = 22, blue; prog, *N* = 23, pink) at first (top left panel) and second (top right panel) measurements (*p* < 0.05; Wilcoxon test for independent samples; FDR corrected; *N_tests_* = 32). Within-cohort differences (*p* < 0.05; Wilcoxon test for paired samples; FDR corrected; *N_tests_* = 32) between first (thick-line) and second (thin-line) measurement for stable (bottom left panel) and progressive (bottom right panel) subcohorts. Shading describes bootstrapped (*n* = 10,000) 95% confidence intervals. ***B***, Percentage of parcels that show either positive or negative difference from first to second measurement in DFA exponents. ***C***, The data for fE/I, as in ***A***. ***D***, Data for fE/I as in ***B***. ***E***, Left, Correlation (Pearson’s *r*) between alpha band (7–12 Hz) DFA exponents, averaged across parcels for each individual, and MMSE scores pooled for stable (blue) and progressive (pink) subcohorts, and first and second measurements. Right, The data for fE/I values.

To further test whether fE/I values and DFA scaling exponents contained information about disease progression within individuals, we analyzed a longitudinal dataset of MCI participants. After assessing their clinical status during follow-up, MCI participants were divided into the stable and progressive groups depending on whether their condition had remained stable or progressed to dementia (see details in Materials and Methods). DFA exponents in frequencies 7–9 Hz and 30–35 Hz were significantly (*p* < 0.05; Wilcoxon test for independent samples; FDR corrected; *N_tests_* = 32) attenuated in the progressive relative to the stable subcohort already at the first measurement (Fig. 6*A*, top left). Consistent with cross-sectional data results, attenuation of DFA exponents were spectrally extended (8–15 Hz) at the follow-up second measurement (Fig. 6*A*, top right; *p* < 0.05; Wilcoxon test for independent samples; FDR corrected; *N_tests_* = 32). For the stable subcohort, the DFA exponents remained stable from the first to the second measurement and were significantly (*p* < 0.05; Wilcoxon test for paired samples; FDR corrected; *N_tests_* = 32) increased only in the high-gamma band at the whole brain (Fig. 6*A*, bottom left) and at the parcel level (Fig. 6*B*). In contrast, DFA exponents for the progressive subcohort were larger (*p* < 0.05; Wilcoxon test for paired samples; FDR corrected; *N_tests_* = 32) at second relative to the first measurement for frequencies above 15 Hz both at the whole brain level (Fig. 6*A*, bottom right) and at the parcel level (Fig. 6*B*). There were no significant differences in fE/I between the cohorts at the first measurement while at the second measurement, the fE/I values were elevated in the progressive cohort (Fig. 6*C*, top right; *p* < 0.05; Wilcoxon test for independent samples; FDR corrected; *N_tests_* = 32). The fE/I values differed also between the first and second measurements only slightly for stable (Fig. 6*C*, bottom left) and more for the progressive (Fig. 6*C*, bottom right) cohort.

Importantly, the alpha-band DFA exponents (Fig. 6*E*, left), but not the fE/I values (Fig. 6*E*, right), pooled across subcohorts and measurements predicted the global cognitive status as measured with MMSE scores, which both demonstrates the overall functional significance of the critical brain dynamics studied here and shows that deterioration in brain criticality predicts individual cognitive decline.

### Machine learning-based classification of dementia stage

**Figure 7.**
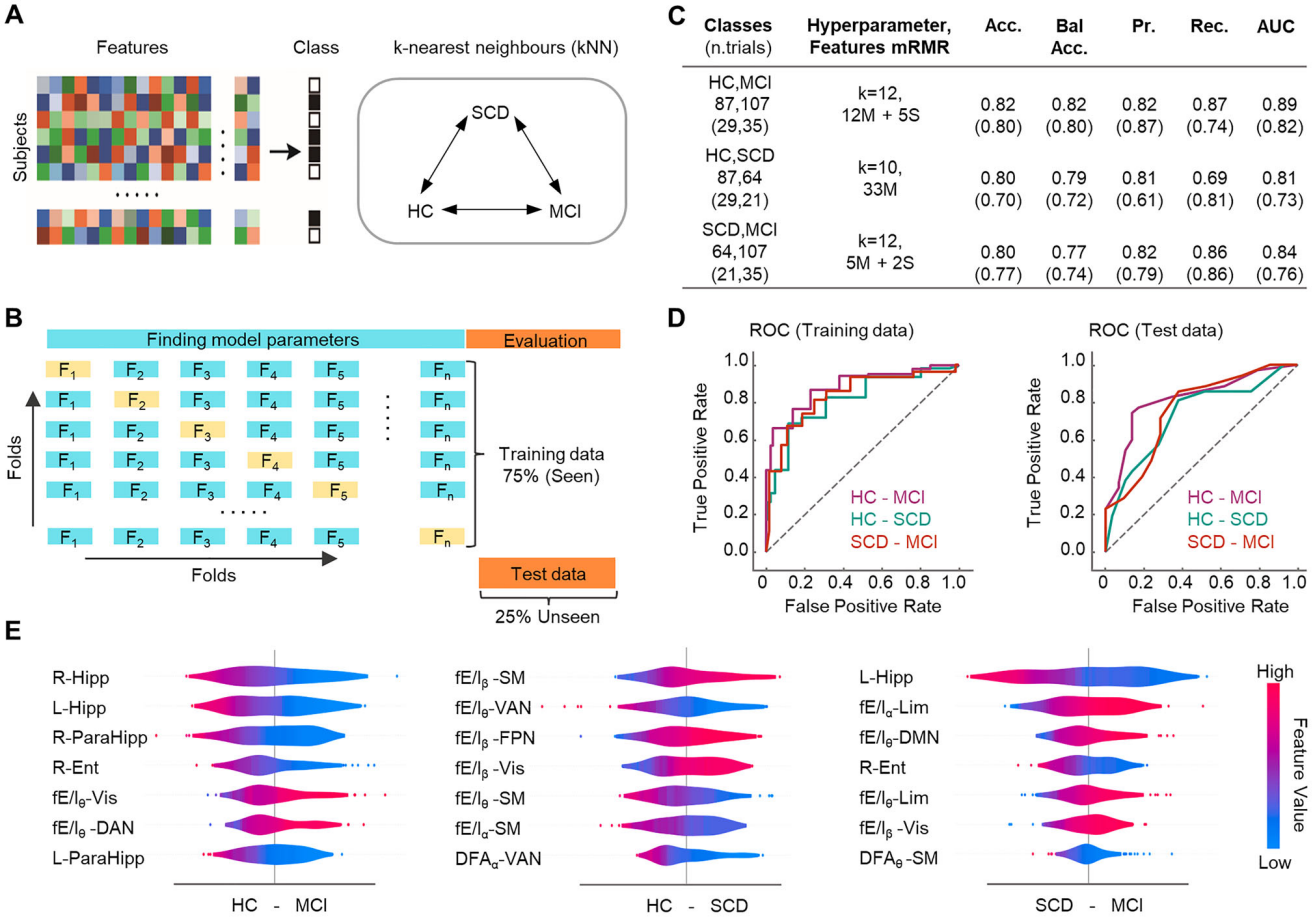
Decoding early stages of AD from MEG data. ***A***, Left, An illustration of the feature matrix, which include functional MEG (LRTCs and fE/I) and structural (MTL volumes) features with the class labels. Right, three separate kNN classifiers were used to classify HC–MCI, HC–SCD, and SCD–MCI cohorts with separate training and test sets. ***B***, The schematic of the approach. First, 75% of the data (training set) was used for the estimation of model parameters [*k* (hyperparameter) and *Nf* (number of predictive features)] and its performance evaluation using leave-one-out cross-validation. Second, remaining 25% of the data (test set) was used to test the classification performance of selected model in the first step. ***C***, Classification performances for the training and validation sets (parenthesis). Accuracy (Acc), balanced accuracy (Bal Acc), precision (Pr), recall (Rec), and area under the curve (AUC). ***D***, ROC curves for training (left) and test (right) sets. ***E***, Seven features ranked by importance (top to bottom) based on their predictive power on the model performance (HC–MCI, left; HC–SCD, middle; SCD–MCI, right). To the right are the SHAP values reflecting the impact of each feature on the model output (i.e., their predictive power). The color code shows whether high (red) or low (blue) values of the feature predict the target category. For example, in the HC–MCI summary plot, right hippocampus volume is the most predictive feature of the target category: HC, and the color code shows that high values of that feature predict HC. Abbreviations: L, left; R, right; Hipp, hippocampus; ParaHipp, parahippocampus; Entorhinal, entorhinal cortex; θ, theta-band; α, alpha band; β, beta band; DMN, default-mode network; SM, sensorimotor network; Vis, visual system; FPN, frontoparietal network; Lim, limbic network; VAN, ventral attentional network; DAN, dorsal attentional network.

Finally, we investigated whether DFA and fE/I measures would allow accurate classification of the dementia stage. To this end, we used independent *k*-nearest neighbors (kNN) classifiers to dissociate the cohorts in this study (HC vs MCI, HC vs SCD, and SCD vs MCI) based on neurophysiological (DFA, fE/I) and structural (MTL volumes) features (Fig. 7*A*). Minimal-optimal features were selected by an mRMR algorithm. Leave-one-out cross-validation was used to estimate the performance of the models using 75% of the data for training (seen). The remaining 25% of the data (unseen) was used for testing (Fig. 7*B*). Both the optimally selected features (fn) and *k* differed for each diagnostic condition: fn = 17 and *k* = 12 for HC–MCI, and fn = 33 and *k* = 10 for HC–SCD, and fn = 7 and *k* = 12 for SCD–MCI. The classification yielded predictions of diagnostic categories of 81.67 and 75.67% (mean accuracy across all binary problems) using training and test sets, respectively (Fig. 7*C*). The ROC curves endorsed the between-categories discriminative ability of the classifiers, with a mean AUC as high as 84.67 and 77% for training and test data, respectively (Fig. 7*D*). Importantly, our model performance metrics—precision (training data, 81 and 82%, and test data, 79 and 61%) and recall (training data, 86 and 69%, and test data, 86 and 81%)—show that SCD individuals could be discriminated from both MCI and HC, respectively.

We used SHAP values to indicate the importance of each feature for classification. MTL volumes were the most relevant features in the classification of MCI versus HC and MCI versus SCD. In contrast, the most relevant features for the classification of SCD from HC were mean fE/I and DFA values (Fig. 7*E*), whereas MTL volumes had only low predictive power (Table 2). We further replicated the classifications by both fixing the number of features to 10 and using the support vector machine (SVM) learning method. We achieved similar classification performances (Table 2), demonstrating that results were robust against chosen methodology.

## Discussion

Healthy brain function is possible only near a net balance of functional excitatory and inhibitory influences. Shifts away from this E/I balance through disproportionate inhibition or excitation are thought to play a mechanistic role in various brain diseases, e.g., via altered functional connectivity (Uhlhaas and Singer, 2012) and critical brain dynamics (Amil and Verschure, 2021). We addressed whether shifts in E/I balance toward excessive excitation and corresponding shifts in the operating point of neuronal dynamics toward supercriticality could underlie dementia progression. Here, using measures of critical brain dynamics and resting-state MEG data from a large cross-sectional cohort of (*N* = 343) of individuals with SCD, MCI, and HC as well as from a longitudinal cohort (*N* = 45) of MCI patients, we found several lines of new evidence for that progression of dementia from SCD to MCI and to AD is characterized by an incremental shift from the brains operating with inhibition-dominated or balanced dynamics (Priesemann et al., 2014; Fuscà et al., 2023) toward operation in an excitation-dominated state on the supercritical side of an extended critical regime (Fuscà et al., 2023).

Due to the lack of direct means for measuring the E/I ratio from noninvasive human electrophysiological recordings, experimental evidence supporting the hypothesis of continuous excessive excitation in dementia disease progression has remained scarce. Attenuated LRTCs have previously been observed in AD (Montez et al., 2009; Van Nifterick et al., 2023). The present findings extend this prior art and reveal that both the preclinical subjective symptoms (in SCD) and the first clinical indications (at MCI) of dementia are already characterized by salient brain criticality abnormalities. Multiple recent frameworks of AD (Zott et al., 2018; Lam and Shafi, 2022; Giorgio et al., 2023), including the “X model” (Pusil et al., 2019), build on the hypothesis that a shift in E/I balance toward excessive excitability leads to excessive neuronal synchronization in prodromal AD patients. Converging theoretical (S. Palva and Palva, 2012), computational modeling (Bruining et al., 2020), and MEG (Fuscà et al., 2023), evidence suggests that individual levels of neuronal synchronization are regulated by the individual operating point along the critical regime. We thus propose that progressive shifts of the individual operating points toward the supercritical phase could lead to progressive emergence of hypersynchronization frequently observed both in human studies and in animal models of AD (Busche and Konnerth, 2016; de Haan et al., 2017; López-Sanz et al., 2017; Babiloni et al., 2020; Maestú and Fernández, 2020).

Disease progression from SCD to MCI was associated with alpha to beta frequency changes in fE/I and LRTCs as well as with anatomical expansion of the changes in neuronal dynamics. Here we demonstrate that measures of critical dynamics (LRTCs, fE/I) enable the accurate classification of individuals with SCD while previous studies have demonstrated that reduced oscillatory power (Jiao et al., 2023) and functional connectivity (Maestú et al., 2015) allow accurate classification of individuals only with dementia progression to MCI. As found previously (Visser et al., 2002), MTL atrophy yielded best classification accuracy for the individuals with MCI. Future studies for developing mechanistic biomarkers based on functional MEG data may enable accurate diagnosis of early-stage AD, to whom traditional diagnosis might be challenging due to high cognitive reserve (Lojo-Seoane et al., 2018).

Surprisingly, the best classification accuracy of early dementia (SCD) was obtained with fE/I albeit the changes in LRTCs across dementia progression were more robust and systematic. The good classification accuracy might be due to the seemingly nonlinear dynamics of fE/I along dementia progression. Using longitudinal MCI cohort, which constitutes a gold standard for AD diagnosis (Jack et al., 2018), we further demonstrated that progressive MCI cohort exhibited larger changes compared with stable MCI cohort. The longitudinal increase in LRTCs and fE/I in progressive MCI patients, the weak albeit nonsignificant suppression of fE/I in SCD, and the strong predictive power of fE/I to the accurate classification of dementia stage are seemingly surprising but align with the nonlinear U-shaped dynamics of brain criticality and the non-monotonic path highlighted by the X model (Pusil et al., 2019). In addition, albeit hypersynchronization is characteristic feature of AD, some studies also report hyposynchronization (Cuesta et al., 2022; Ranasinghe et al., 2022; Schoonhoven et al., 2022), which might reflect either an advanced disease stage with a network breakdown (Pusil et al., 2019) possibly due to tau pathology (Canuet et al., 2015) or a different nonepileptiform AD phenotype (Maestú et al., 2021). Besides E/I ratio, also structural connectivity, synaptic alterations, and other biological mechanisms contribute to the control parameters of critical brain dynamics (Moretti and Muñoz, 2013; Villegas et al., 2014). Taken together the emergence of brain atrophy (Zhang et al., 2021), white matter loss (Festa et al., 2023), and changes in interneuron circuitry (Garcia-Marin et al., 2009) in later stages of AD, also evident in the present study, the nonlinear changes in LRTCs and fE/I might be due to complex interactions between E/I ratio and changes in brain structure and neurobiology. These results suggest a severe pathological breakdown of brain dynamics as individuals progress toward AD. These together point toward progressive changes in brain dynamics in early AD continuum.

Importantly, we found correlations between the breakdown of LRTCs and cognitive performance scores. Given that neuronal oscillations regulate information processing in distributed neuronal circuits (Singer, 1999; Hahn et al., 2019; Arnulfo et al., 2020; Schirner et al., 2022; Vinck et al., 2023) thereby serving fundamental functional roles in variety of human sensory and cognitive functions (S. Palva and Palva, 2012; Thut et al., 2012), shifts toward supercritical dynamics are likely to lead to cognitive deficits characteristic to early-stage AD via their relationship with synchronization (Fuscà et al., 2023). Given that supercritical brain dynamics is associated with overly amplified signal propagation predisposing to epileptogenic activity (Meisel et al., 2015), we propose that progressive shift in the operating point toward supercritical phase could also explain the epileptiform phenotype of AD (Busche and Konnerth, 2016; de Haan et al., 2017; López-Sanz et al., 2017; Babiloni et al., 2020; Maestú and Fernández, 2020; Ranasinghe et al., 2022), observed also in subcohort of the present study (Cuesta et al., 2022). These progressive changes toward supercriticality could thus lead to characteristic symptoms of AD disease progression. Albeit we used fE/I and LRTCs to assess brain operating point, other measures can also be used to index interindividual neuronal variability, critical dynamics, and E/I balance (Ahmad et al., 2022).

In resume, our findings provide a new perspective for early AD, whereby AD disease progression can be understood in the extended brain criticality framework. The findings advanced here could significantly enhance understanding of AD pathophysiology with a focus on early diagnosis and open new avenues for pharmacological and other interventions targeted at recovering and sustaining the E/I balance, thereby preventing aberrant brain dynamics and excitotoxicity.
